# One- and two-stage surgical revision of infected elbow prostheses following total joint replacement: a systematic review

**DOI:** 10.1186/s12891-019-2848-x

**Published:** 2019-10-22

**Authors:** Setor K. Kunutsor, Andrew D. Beswick, Michael R. Whitehouse, Ashley W. Blom

**Affiliations:** 1 National Institute for Health Research Bristol Biomedical Research Centre, University Hospitals Bristol NHS Foundation Trust and University of Bristol, Bristol, UK; 2 Translational Health Sciences, Bristol Medical School, Musculoskeletal Research Unit, University of Bristol, Learning & Research Building (Level 1), Southmead Hospital, Bristol, BS10 5NB UK

**Keywords:** Prosthetic joint infection, Elbow replacement, Revision, One-stage, Two-stage, Systematic review

## Abstract

**Background:**

Prosthetic joint infection (PJI) is a challenging complication of total elbow replacement (TER). Potential surgical treatments include one- or two-stage revision; however, the best treatment for elbow PJI is not clearly defined. We conducted a systematic review in accordance with PRISMA guidelines to compare the clinical effectiveness of one- and two-stage revision surgery for elbow PJI using re-infection (recurrent and new infections) rates; mortality; clinical measures of function, pain, and satisfaction; and non-infection related adverse events.

**Methods:**

MEDLINE, Embase, Web of Science, and The Cochrane Library were searched up to June 2019 to identify observational cohort studies and randomised controlled trials (RCTs) that had recruited patients with elbow PJI following TER and treated with one- or two-stage revision. Of 96 retrieved articles, 2 one-stage and 6 two-stage revision studies were eligible. No RCT was identified. Arcsine transformation was used in estimating rates with 95% confidence intervals (CIs).

**Results:**

*Staphylococcus aureus* was the most common causative organism for PJI of the elbow (24 of 71 elbow PJIs). The re-infection rate (95% CI) for one-stage (7 elbows) ranged from 0.0% (0.0–79.3) to 16.7% (3.0–56.4) and that for two-stage revision (87 elbows) from 0.0% (0.0–49.0) to 20.0% (3.6–62.4). Non-infection related adverse event rate for one-stage (based on a single study) was 16.7% (3.0–56.4) and that for two-stage ranged from 11.8% (4.7–26.6) to 20.0% (3.6–62.4). There were no mortality events recorded following one- or two-stage revision surgery and postoperative clinical measures of function, pain, and satisfaction could not be effectively compared because of limited data.

**Conclusions:**

No strong conclusions can be drawn because of limited data. The one-stage revision may be potentially at least as clinically effective as two-stage revision, but further data is needed. There are clear gaps in the existing literature and studies are urgently warranted to assess the clinical effectiveness of one- and two-stage revision strategies for PJI following TER.

**Systematic review registration:**

PROSPERO 2018: CRD42018118002.

## Background

Common complications following total elbow replacement (TER) include implant loosening, periprosthetic fracture, implant failure, triceps insufficiency, nerve palsy, and prosthetic joint infection (PJI) [1]. Compared to lower extremity joint replacements, relatively few elbow replacements are performed. In 2017, as recorded in the National Joint Registry for England, Wales and Northern Ireland and the Isle of Man, approximately 100,000 joint replacements were performed each in knees and hips; whereas only 612 elbow replacements were performed [2]. However, it has been reported that the proportion of complications associated with elbow replacements is greater than that for hip or knee replacements [3].

Prosthetic joint infection (PJI) is a potentially devastating complication of TER and compared with hip or knee replacement, TER is associated with a higher incidence of PJI which affects between 1 to 12% of patients [4–6]. PJI after TER is associated with significant morbidity [7] as well as increased costs to the healthcare system [8]. Treatment of elbow PJI is a challenging task [9] and the key goals are clearing infection, retaining maximum joint function, and pain relief. Treatment options for elbow PJI include debridement, treatment with antibiotics and retention of the prosthesis (DAIR); resection arthroplasty; and one- or two-stage revision [10]. Resection arthroplasty is generally considered a salvage procedure and used as a last resort in refractory PJI after TER or in patients for whom loss of elbow function is of less concern [11, 12]. The best treatment for elbow PJI is not clearly defined as choices of treatment strategy are generally based on the treating surgeon’s experience and evidence derived from studies of PJI treatment in hip and knee replacement [13]. Based on existing data, the two-stage revision strategy appears to be the most commonly used treatment option for elbow PJI [10]. The one-stage revision strategy may be a putative alternative treatment. An extensive body of evidence suggests that one- and two-stage revision strategies for hip, knee and shoulder PJI are clinically comparable [14–17]; however, the data are sparse and conflicting on the role of these strategies for treating infected elbow prostheses. To our knowledge, no study has yet reviewed the existing evidence by comparing results of published studies that have reported clinical outcomes on any of these two revision strategies. We are also not aware of any randomised controlled trial (RCT) that has compared the clinical effectiveness of the two revision strategies.

To clarify the existing evidence, we conducted a systematic review to compare the clinical effectiveness of the one- and two-stages revision strategies for elbow PJI using infection control as the primary outcome. Secondary objectives included (i) comparing the effectiveness of the two revision strategies based on other clinical outcomes which include mortality; validated measures of function, pain, and satisfaction; as well as non-infection related adverse events and (ii) to explore any gaps in the evidence base.

## Methods

### Data sources and search strategy

We registered this review in the PROSPERO prospective register of systematic reviews (CRD42018118002). The review was based on a protocol which was predefined and performed following PRISMA and MOOSE guidelines [18, 19]. We systematically searched MEDLINE, Embase, and The Cochrane library from inception to 25 June 2019 for longitudinal observational studies and RCTs that reported on infection control and/or other clinical endpoints after one- or two-stage surgical revision of an infected elbow prostheses. The computer-based searches employed a combination of free and MeSH search terms and key words related to the intervention, population and outcomes. Only human studies were searched for, with no restrictions placed on language. The full search strategy is reported in Additional file 1: Table S1. The retrieved citations were initially screened based on their titles and abstracts to assess their potential for inclusion, after which we retrieved potentially eligible articles for full text evaluation. Evaluation of full texts was conducted by two independent authors (S.K.K., A.D.B.) based on the inclusion criteria and any disagreements regarding whether an article should be included or not was discussed, with involvement of a third author (M.R.W) to reach a consensus. We also scanned reference lists of relevant articles (including reviews) for studies missed by the original search. The “cited by” function in Web of Science was used to check for citations of key studies.

### Eligibility criteria

Studies were eligible for inclusion if they (i) were longitudinal observational studies or RCTs that included patients with infected elbow prostheses following TER and were managed by a one- or two-stage revision strategy and (ii) were followed up post-operatively for re-infection (which was defined as recurrence of infection by the same organism(s) and/or re-infection with a new organism(s)) and/or other clinical outcomes such as (a) function [as measured by the Mayo elbow performance score (MEPS); flexion-extension range of motion; and triceps function]; (b) pain; (c) satisfaction; or (d) non-infection related complications (such as implant failure, periprosthetic fracture, loosening, haematoma, postoperative instability, nerve entrapment, and triceps insufficiency).

### Data extraction and quality assessment

One author (S.K.K.) initially extracted the data using a data collection form which was standardised for this purpose. A second author (A.D.B) checked these data independently with the information in the original articles. Any disagreements were discussed and a third author (M.R.W) was involved to reach a consensus. We extracted the following pieces of information: Author and year of publication, study design, study location, mean or median age at baseline, proportion of male participants, type of revision surgery, revision surgery characteristics, duration of follow-up after revision surgery, number of re-infections after revision surgery, other clinical outcomes, and adverse events. If information about the same study was published twice or more often, we used the most recent publication or the one with most up to date information. The methodological quality of included studies was assessed using the Methodological Index for Non-Randomised Studies (MINORS), a well-established validated instrument designed for assessing the quality of non-randomised studies in surgery [20] and which has been described in previous published papers [14, 15]. Briefly, this tool uses eight pre-defined factors which include: a clearly reported aim, inclusion of consecutive patients, data collected in a prospective manner, endpoints reflecting the aim of the study, study endpoints assessed in an unbiased manner, follow-up duration which is appropriate to the aim of the study, less than 5% loss to follow-up, and prospective calculation of the study sample size. For each of the domains, the tool assigns a score of 0 for “not reported”, 1 for “reported but inadequate”, or 2 for “reported and adequate”. These are then added into a total score. A score of 16 is regarded as the global ideal score.

### Data analysis

The re-infection rate, which was the primary outcome, was computed from the number of re-infections within the follow-up period following elbow revision surgery divided by the total number of participants with PJI or number of elbow joints with PJI. Re-infection rates with 95% confidence intervals (CIs) were estimated across the studies by employing the Freeman-Tukey variance stabilising double arcsine transformation [21]. Details of the method have been described previously [14, 15]. Given the limited data, a pooled analysis was not performed. Non-infection related adverse event rates (computed from the number of adverse events or complications within follow-up period following elbow revision surgery divided by the total number of participants with PJI or number of elbow joints with PJI) with 95% CIs were also estimated across studies. Stata MP 16 (Stata Corp, College Station, Texas, USA) was employed for all statistical analyses.

## Results

### Study identification and selection

**Fig. 1:**
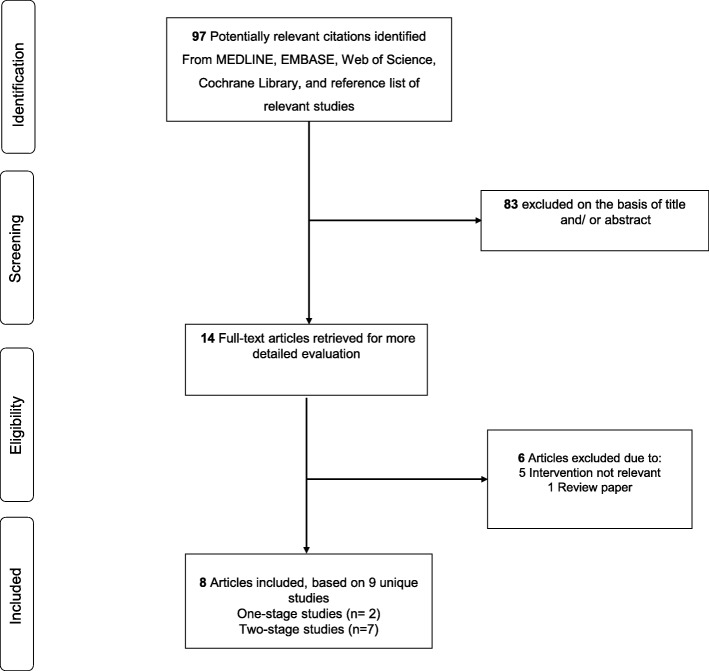
PRISMA flow diagram

Of 97 records retrieved from the search, we excluded 83 articles based on titles and abstracts. On reviewing the full texts of the remaining 14 articles, we excluded a further 6 articles because (i) intervention was not relevant (*n* = 5) or the article (ii) was a review paper (*n* = 1). This left 8 articles eligible for inclusion in the review (Fig. 1; Table 1) [12, 13, 22–27].

**Table 1:** Summary characteristics of included studies

	One-stage revision	Two-stage revision
Eligible studies		
Total number of studies included	2	7
Participants		
Total number of participants	7	87
Total number of re-infections	1	11
Median (IQR) age (years)	64.9 (62.7–67.0)	64.7 (59.7–65.0)
Median (IQR) males (%)	0.0 (0.0–0.0)	31.0 (29.5–35.8)
Location		
Europe	2 (7)	4 (59)
North America	–	3 (28)
Asia	–	–
Study and surgery characteristics		
Median (IQR) time from index surgery to infection diagnosis (months)	40.5 (40.5–40.5)	38.0 (24.0–51.9)
Median (IQR) duration of infection symptoms (days)	–	60.4 (60.4–60.4)
Median (IQR) time from index surgery to revision surgery (months)	–	57.1 (48.2–66.0)
Median (IQR) from infection diagnosis to revision surgery (months)	–	15.0 (15.0–15.0)
Median (IQR) interval between stages (months)	NA	6.1 (4.7–7.7)
Median (IQR) follow-up (years)	5.4 (4.0–6.8)	4.1 (3.0–4.3)
Median (IQR) duration of antibiotics (days)	–	30.5 (11.3–49.7)
Median (IQR) duration of IV antibiotics (days)	37.2 (37.2–37.2)	8.8 (8.8–8.8)
Median (IQR) duration of oral antibiotics (days)	–	2.5 (2.5–2.5)
Methodological quality (IQR)	10.5 (10.0–11.0)	10.0 (10.0–11.0)
Baseline clinical characteristics		
Median (IQR) Range of motion (°)	–	72.0 (50.0–94.0)
Median (IQR) Extension	–	36.3 (36.3–36.3)
Median (IQR) Flexion	–	101.3 (101.3–101.3)
Median (IQR) MEPS	–	28.0 (22.9–52.5)
Median (IQR) Pain score	–	15.0 (15.0–15.0)

*IQR* Interquartile range, *IV* Intravenous, *MEPS* Mayo elbow performance score, *NA* Not applicable; values are number of studies (number of participants) unless stated otherwise

### Study characteristics and study quality

Table 1 provides a summary of baseline characteristics of one- and two-stage revision studies which were eligible. Details on individual study baseline characteristics and methodological quality are reported in Table 2. Of the 8 eligible articles, 6 articles were based on two-stage revision; 1 article evaluated the one-stage revision; and 1 article evaluated both one- and two-stage revision strategies. Overall, there were 9 unique studies comprising 94 elbow joints revised for PJI (92 participants) and 13 re-infections. All included studies retrospectively analysed data based on observational cohort designs or case series. The most common surgical indication for the index TER was rheumatoid arthritis. We did not identify any clinical trials comparing both revision strategies. Studies were carried out in Europe (UK, Germany, and Switzerland) and North America (United States of America). Baseline study level surgery and clinical characteristics could not be compared between the two revision strategies because of the limited number of studies and outcome measures for one-stage revision. Studies reported the diagnosis of PJI in a variety of ways, but was mainly based on the presence of one or more of the following criteria: (i) clinical, haematological and radiological assessments suggesting the diagnosis with persistent swelling and inflammation, high blood indices (such as white cell count, C-reactive protein, or erythrocyte sedimentation rate) and progressive radiolucent lines; (ii) positive results of microbiological culture from preoperative elbow joint aspirate, intraoperative periprosthetic tissue, or sonication fluid of the removed plant; (iii) visible purulence of a preoperative aspirate or intraoperative periprosthetic tissue; (iv) wound findings such as the presence of a sinus tract communicating with the prosthesis; and (v) pathological findings on tissue sections. *Staphylococcus aureus (S. aureus)* was reported as the most common causative organism for elbow PJI in the majority of eligible studies that provided these data (24 out of 71 elbow PJIs). Intravenous flucloxacillin and rifampin were the most common antibiotics administered following revision surgery. The methodological quality scores of studies included in the review ranged from 9 to 11.

**Table 2:** Baseline characteristics of individual studies included in review

Lead Author, Publication Date (Reference No.)	Location	Year of study	Mean/median age (years)	% male	Indication for index TER	Mean/median interval between stages (months)	Common infecting organism	Common IV ATBs given after revision surgery	Follow up Mean/median (years)	No. of re-infections	No. of deaths	No. of non-infection related adverse events	No. of participants	No. of elbows joints	Quality score
One-stage															
Gille, 2006 [23]	Germany	1978–1999	62.7	0.0	RA (100%)	NA	*S. aureus*	Flucloxacillin	6.8	1	0	1	5	6	10
Spormann, 2012 [24]	Switzerland	1994–2007*	67.0	0.0	NR	NA	*CNSA*	Oral ciprofloxacin/rifampin given	4.0	0	NR	NR	1	1	11
Two-stage															
Yamaguchi, 1998 [12]	USA	1981–1994	53.8	40.0	RA (80%); OA (20%)	3.7	*S. aureus; S. epidermidis*	None given	4.1	1	0	1	5	5	11
Yamaguchi, 1999 [22]	USA	1981–1994	59.7	28.6	RA (85.7%); PTA (14.3%)	NR	*S. aureus; S. epidermidis*	NR	4.3	1	0	4	7	7	10
Achermann, 2011 [13]	Switzerland	1994–2007	NR	NR	RA; OA	5.5	NR	Flucloxacillin/rifampin	NR	0	NR	NR	2	2	9
Spormann, 2012 [24]	Switzerland	1994–2007	54.5	100	RA; PTA; OA; psoriasis	NR	*CNSA*	Flucloxacillin/rifampin	6.4	0	NR	NR	4	4	11
Peach, 2013 [25]	UK	1998–2010	65.0	30.3	RA; fracture; OA; PTA	9.0	*CNSA*	Cefuroxime	4.3	4	0	2	33	34	11
Rudge, 2018 [26]	UK	2009–2014	64.7	31.6	RA; PTA; fracture; OA	6.4	*S. aureus*	Tazocin, teicoplanin, amikacin	3.0	3	0	9	19	19	10
Zmistowski, 2018 [27]	USA	2001–2016	NR	NR	PTA; RA; Unknown; fracture	5.7	NR	NR	3.0	3	0	3	16	16	10

*ATBs* Antibiotics, *CNSA* Coagulase negative *Staphylococcus aureus*, *IV* Intravenous, *NA* Not applicable, *NR* Not reported, *OA* Osteoarthritis, *PTA* Post-traumatic arthritis, *RA* Rheumatoid arthritis, *S* Staphylococcus; *, for all participants included in the series

### Revision strategy and re-infection

**Fig. 2:**
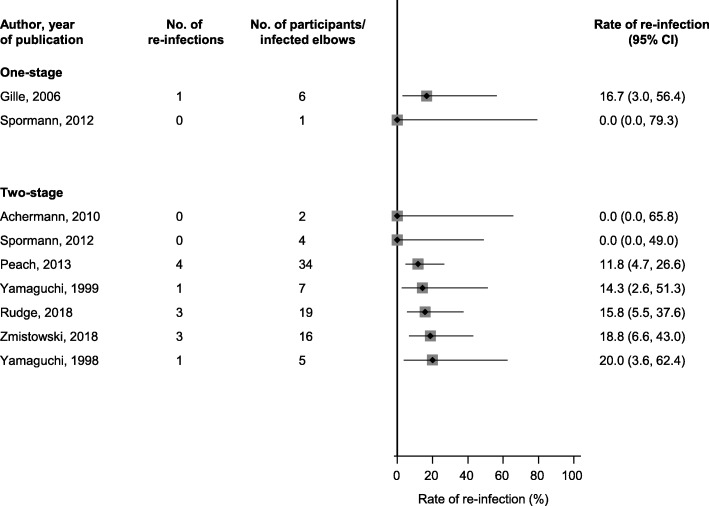
Rates of re-infection in infected elbow prostheses treated by one- and two-stage revision. CI, confidence interval (bars)

Two studies were reported to have evaluated the one-stage revision strategy and comprised of 7 elbow joints revised for PJI (6 participants) and 1 re-infection (Tables 1 and 2). The re-infection rate ranged from 0.0% (95% C: 0.0–79.3) to 16.7% (95%CI: 3.0–56.4) over a weighted mean follow-up period of 6.4 years (Fig. 2).

Seven studies comprising of 87 elbow joints revised for PJI (86 participants), reported 12 re-infections following two-stage surgical revision (Tables 1 and 2). The re-infection rate ranged from 0.0% (95% CI: 0.0–49.0) to 20.0% (95% CI: 3.6–62.4) over a weighted mean follow-up period of 3.7 years (Fig. 2).

### Other post-operative clinical outcomes

Of the six studies (1 one-stage and 5 two-stage studies) that reported on mortality outcomes, none reported any mortality event associated with the revision strategy (Table 2). The same studies reported on non-infection related adverse events following revision surgery and these included explantation; revision; triceps weakness, insufficiency, or rupture; ulnar fracture; ulnar nerve neuropraxia; skin breakdown; humeral component loosening; and non-union (Table 2). The non-infection related adverse event rate for one-stage which was based on one study was 16.7% (95% CI: 3.0–56.4) and that for two-stage revision ranged from 11.8% (95% CI: 4.7–26.6) to 20.0% (95% CI: 3.6–62.4) over a weighted mean follow-up period of 3.7 years (Additional file 1: Figure S1).

Measures of function and pain between both revision strategies could not be compared using statistical tests because data from one-stage revision was based on limited data points (Table 3). However, median computed values for range of motion, flexion, and MEPS seemed to be better in the two-stage revision group compared with the one-stage group; whereas extension was better in one-stage revision. In 2 two-stage studies that reported on measures of satisfaction, 4 out of 4 patients reported satisfaction with the outcomes in one study and in the other, 6 out of 7 patients were satisfied with their outcomes. One one-stage study reported on this outcome and indicated 4 out of 6 patients as being satisfied with the outcomes post-surgery.

**Table 3:** Post-operative clinical outcomes following one- and two-stage revision strategies

	One-stage revision	Two-stage revision
Median (IQR) Range of motion (°)	90.0 (80–90)	104.2 (97.0–111.4)
Median (IQR) Extension	35.0 (20.0–40.0)	18.8 (18.8–18.8)
Median (IQR) Flexion	120.0 (100.0–130.0)	129.6 (128.0–131.3)
Median (IQR) MEPS	67.6 (50.0–80.0)	83.4 (77.1–90.4)
Median (IQR) Pain score	–	38.6 (38.6–38.6)

*IQR* Interquartile range, *MEPS* Mayo elbow performance score

## Discussion

In this literature-based systematic review, **t**he data suggests that one-stage revision may be associated with lower re-infection and non-infection related adverse event rates compared with the two-stage strategy, although the 95% confidence intervals overlapped and the estimated rates were based on very limited data. Measures of function, pain, and satisfaction could not be compared effectively because of limited data. However, the data suggested that range of motion, flexion, and MEPS were improved in the two-stage revision group compared with the one-stage group. These findings reflect evidence observed in other joints showing that two-stage revision may potentially be associated with improvement in function, but lower rates of infection eradication compared with one-stage revision [15, 16]. The findings cannot be compared with previous work, given this is the first ever systematic review to compare the clinical effectiveness of one- and two-stage revision strategies for the management of infected elbow prosthesis.

Based on the limited data, it is difficult to make any conclusions on which revision strategy is more clinically effective. However, it appears the one-stage revision may be potentially at least as effective compared with the two-stage revision strategy, given the low re-infection and adverse event rates. This review has also identified large gaps in the existent literature – it appears that though TERs are associated with higher incidence of PJIs compared with hip or knee replacements [4–6], published series on the use of the two most established PJI treatment strategies are non-existent for elbows. It is obvious that the paucity of data on treatment of elbow PJI reflects the lower incidence of TER utilization [27] compared to hip and knee replacements. This also raises the question on whether infections in elbow arthroplasty run a completely different course clinically and hence can’t be treated with one- or two-stage revision surgery in the same way as other joint replacements? As a result of the thin soft tissue envelope of the elbow joint, it is particularly susceptible to infection and this can be worsened by inflammatory conditions (eg, arthritis), medications used for treating these inflammatory diseases and trauma [1]. Majority of elbow infections are caused by skin bacteria such as *S. aureus* and *S. epidermidis.* In contrast to PJIs of the hip and knee joints, prosthetic elbow infections rarely involve systemic symptoms such as fever or malaise [4, 9]. Furthermore, unlike tests for diagnosing prosthetic hip and knee infections, the diagnostic utility of blood testing and joint aspiration for PJI of the elbow is not well established [10]. In a review of the diagnosis and management of prosthetic elbow infection, Somerson and colleagues note that given the lack of objective criteria to diagnose PJI of the elbow, a high index of clinical suspicion is required in addition to knowledge of risk factors and discriminating interpretation of laboratory tests [10]. This especially makes the diagnosis and treatment a challenge. We noted that the average interval between stages in two-stage revision of infected elbow joints was longer (6.1 months) compared to other joints (3–4 months) [15, 16]; indeed, except for one study, the majority of studies reported an average of more than 5 months duration between stages. This observation may reflect a more challenging and protracted course of treatment for elbow PJI compared to that of other joints. Although it is clear from the evidence that two-stage revision is currently the gold standard treatment for the management of elbow PJI; there are no clear management guidelines or consensus as to which revision strategy is the most effective because of the very limited data available. Other treatment options for infected elbow prostheses have been reported to be associated with acceptable outcomes and these include DAIR and resection arthroplasty [7, 28]. However, debridement with suppressive antibiotic therapy has been reported to offer benefit only in the early post-operative period [9]. Resection arthroplasty is considered to be a salvage procedure which is only of benefit for frail patients and those in whom elbow function is not a major concern [7, 12]. The two-stage revision strategy is commonly associated with high infection control rates in lower limb replacement [14, 15] and shoulder arthroplasty [16], but it requires two major surgical procedures which usually cause substantial functional impairment and prolonged periods of hospitalisation [29]. It is also associated with high health service costs [8]. Data, generally from hip and knee joints, suggest that the one-stage revision strategy may have several advantages over the two-stage revision which include shorter periods of hospitalisation and antibiotic therapy, better functional results, and significant cost savings [30, 31]. Currently, there is inadequate evidence to show that these advantages may be applicable to elbow joint PJI. Given the significant burden of elbow PJI to the patient, the surgeon and society as a whole, there is a great need for further research in this area to address the existing gaps. We encourage investigators with access to case series on elbow joint PJI treatment to publish their outcomes on follow-up of their patients.

Given the sparse evidence on the topic, our review represents the first attempt at bringing all the evidence together using a systematic approach. The search strategy was comprehensive and involved multiple databases, with manual reference scanning and no language restrictions; which made it unlikely that we had missed any relevant study conducted on the topic. Though the data was limited and sparse on outcome measures, harmonisation to consistent comparisons enabled interpretation of the findings. We took into account the low event rates reported by the majority of the studies. Finally, we conducted a detailed assessment of the methodological quality of the included studies based on a well validated tool. There were important limitations to this review and these were all related to the included studies. The included studies recruited participants between 1978 to 2016; hence given that some of these studies were conducted several decades ago, inclusion of these data may not reflect current standards of practice, as TER implant designs have changed over time [32]. Prosthetic designs and surgical techniques have improved as well as the introduction of newer and more effective antimicrobial therapies, therefore including these older studies could have biased the outcomes. There was a small possibility that two of the two-stage revision studies had overlapping patients [13, 24] and attempts to get the original authors to confirm or refute this proved futile. Whereas some studies did not report the definition of PJI, those reported by other studies varied and these could have biased the findings; however, the majority of studies diagnosed PJI using similar criteria. A robust comparison of all outcomes of interest could not be made between the two revision strategies because of the limited number of published studies and outcome data reported. The sample sizes were small and had very low event rates. These limitations precluded detailed analyses and effective comparisons. The findings therefore need to be interpreted with caution. However, the current findings are timely and relevant because they provide substantial insight on the huge gaps in the existing literature. In the absence of case series to compare the effectiveness of the two revision strategies, there is a potential that data from national joint registries may be useful in answering these questions.

## Conclusions

No strong conclusions can be drawn because of limited data. The one-stage revision strategy may be potentially as clinically effective as the two-stage revision for the treatment of elbow PJI, but further data is required. Remarkable findings are the clear gaps in the existing literature and studies are urgently warranted to evaluate the clinical effectiveness of one- and two-stage revision strategies for treating elbow PJI.

## Supplementary information

**Additional file 1: Table S1.** Literature search strategy.

**Additional file 2: Figure S1.** Rates of non-infection related adverse events in infected elbow prostheses treated by one- and two-stage revision.

## Data Availability

The sources of the data analysed in the study have been detailed in the manuscript.

## Abbreviations

CI: Confidence interval
DAIR: Debridement, treatment with antibiotics and retention of the prosthesis
MEPS: Mayo elbow performance score
MINORS: Methodological Index for Non-Randomised Studies
PJI: Prosthetic joint infection
RCT: Randomised controlled trial
TER: Total elbow replacement
