# Supplementary material for: One- and two-stage surgical revision of infected elbow prostheses following total joint replacement: a systematic review
Source: BMC Musculoskelet Disord. 2019 Oct 22;20:467. doi: 10.1186/s12891-019-2848-x (PMC6806568; doi:10.1186/s12891-019-2848-x)
Supplement: Supplementary file 1 — Additional file 1: Table S1. Literature search strategy. [file 12891_2019_2848_MOESM1_ESM.doc]

**Additional file 1: Table S1.** Literature search strategy

Relevant studies, published before 25 June 2019 (date last searched), were identified through electronic searches not limited to the English language using MEDLINE, EMBASE, Web of Science, and Cochrane databases. Electronic searches were supplemented by scanning reference lists of articles identified for all relevant studies (including review articles), by hand searching of relevant journals and by correspondence with study investigators. The computer-based searches combined search terms related to elbow replacement, periprosthetic joint infection, and revision with focus on one- and two stage surgeries.

1 exp Prosthesis-Related Infections/ or prosthetic joint infection.mp. (11302)

2 exp INFECTION/ (746321)

3 exp Wound Infection/ (44161)

4 exp Surgical Wound Infection/ (34043)

5 surgical site infection.mp. (5752)

6 exp SEPSIS/ (113818)

7 exp ARTHROPLASTY, REPLACEMENT, ELBOW/ (323)

8 exp ELBOW/ (6656)

9 exp Elbow Joint/ (11676)

10 one-stage.mp. (11002)

11 two-stage.mp. (23526)

12 one stage.mp. (11002)

13 two stage.mp. (23526)

14 single-stage.mp. (6629)

15 prosthesis exchange.mp. (28)

16 exchange.mp. (294219)

17 direct exchange.mp. (243)

18 revis$.mp. (175044)

19 revision arthroplasty.mp. (1459)

20 staged revision.mp. (76)

21 exp REOPERATION/ (80111)

22 reimplant$.mp. (7791)

23 1 or 2 or 3 or 4 or 5 or 6 (761093)

24 7 or 8 or 9 (17104)

25 10 or 11 or 12 or 13 or 14 or 15 or 16 or 17 or 18 or 19 or 20 or 21 or 22 (572572)

26 23 and 24 and 25 (70)

27 limit 26 to humans (70)

Each part was specifically translated for searching the other databases (EMBASE, Web of Science, and Cochrane databases)

**Supplementary Figure S1**. Rates of non-infection related adverse events in infected elbow prostheses treated by one- and two-stage revision

CI, confidence interval (bars)
