# Supplementary material for: One- and two-stage surgical revision of infected elbow prostheses following total joint replacement: a systematic review
Source: BMC Musculoskelet Disord. 2019 Oct 22;20:467. doi: 10.1186/s12891-019-2848-x (PMC6806568; doi:10.1186/s12891-019-2848-x)
Supplement: Supplementary file 2 — Additional file 2: Figure S1. Rates of non-infection related adverse events in infected elbow prostheses treated by one- and two-stage revision. [file 12891_2019_2848_MOESM2_ESM.doc]

**Additional file 2: Figure S1**. Rates of non-infection related adverse events in infected elbow prostheses treated by one- and two-stage revision

CI, confidence interval (bars)
